# Case report: Mounded and refractory keratoses (MARK), a novel presentation of pemphigus vulgaris

**DOI:** 10.3389/fmed.2022.1087382

**Published:** 2023-01-10

**Authors:** Stephanie T. Le, Atrin M. Toussi, Jordan E. Nava, Lauren A. Downing, Maxwell A. Fung

**Affiliations:** ^1^Department of Dermatology, University of California, Davis, Sacramento, CA, United States; ^2^Department of Pathology, University of California, Davis, Sacramento, CA, United States

**Keywords:** pemphigus, immunobullous, blistering, pemphigus vegetans, pemphigus vulgaris

## Abstract

Pemphigus vulgaris (PV) is a rare immunobullous disease. Although it classically presents as generalized flaccid blisters affecting the skin and mucosae, atypical cases of PV can be diagnostically challenging. Herein, we report an underrecognized non-blistering manifestation of pemphigus vulgaris, which we call mounded and refractory keratoses (MARK). MARK presents as exuberant scaling plaques on the scalp, often in the skin of color. When MARK features are present, pemphigus vulgaris is prone to misdiagnosis, clinically and histopathologically, leading to delays in appropriate treatment. Specifically, biopsies from these patients may resemble acantholytic dyskeratosis, resulting in initial misdiagnosis. Thus, recognizing this presentation may aid physicians in diagnosing and monitoring the recurrence of pemphigus vulgaris.

## Introduction

Pemphigus vulgaris (PV) is a rare autoimmune disease, classically characterized by flaccid blisters affecting the skin and mucosae (1). PV is driven by pathogenic autoantibodies to desmoglein (Dsg) 3 and Dsg1 (2, 3), resulting in the loss of cellular adhesion and intraepidermal blistering. PV is the most common subtype of pemphigus, accounting for 70% of cases (3). The incidence of PV is approximately 1–5 cases per 100,000 population, most commonly affecting ages 40–60 (4). If untreated, PV is associated with high mortality (5). Classically, PV presents with diffuse flaccid blisters of the skin and oral and pharyngeal mucosa. Herein, we discuss four cases of PV in the skin of color that presented with the underrecognized manifestation of mounded and refractory keratoses (MARK). Biopsies were misinterpreted as an acantholytic dyskeratotic process, either Darier disease or warty dyskeratoma. Despite being a dermatologic disease of historic significance, this particular presentation remains underrecognized.

## Case 1

**FIGURE 1:**
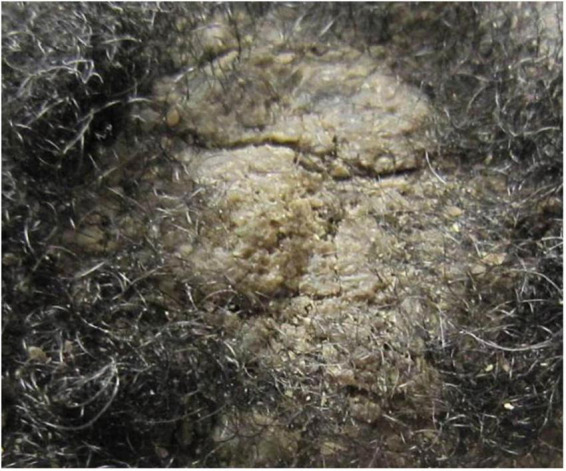
Mounded and refractory keratoses, an atypical presentation of pemphigus vulgaris affecting the scalp. Presentations can include brown–gray mounded scales with or without the crust.

A 19-year-old African American male was referred to the dermatology clinic for the evaluation of a 5-month history of multiple scaly plaques on his scalp. The initial lesion affecting his anterior scalp was reportedly incited by trauma, but the plaques continued to spread to involve his entire scalp and nose. He was also noted to have several fluid-filled lesions. Prior treatments included one 10-day course of doxycycline, with which he noted no improvement. A physical exam revealed dry, matted mounded plaques with powdery scales, some with yellow-green crust, present throughout his entire scalp (Figure 1). Focal areas of alopecia were noted. During the hair pull test, scalp hairs are released with gentle and firm tension at the edges of the focal areas of alopecia. No active oozing or bogginess of the scalp was present. Several areas behind the left ear and on the nose had exuberant mounded scales, many with golden-yellow to brown crusting. Scattered scabbed erosions were present on the chest, arms, and upper back. There were also a few mounded plaques present on his trunk. One clear, fluid-filled vesicle was localized on the left upper back. The clinical differential diagnosis included tinea amiantacea, tinea capitis, and deep fungal infection. The patient was preemptively given a 10-day course of cephalexin and ketoconazole 2% shampoo.

**FIGURE 2:**
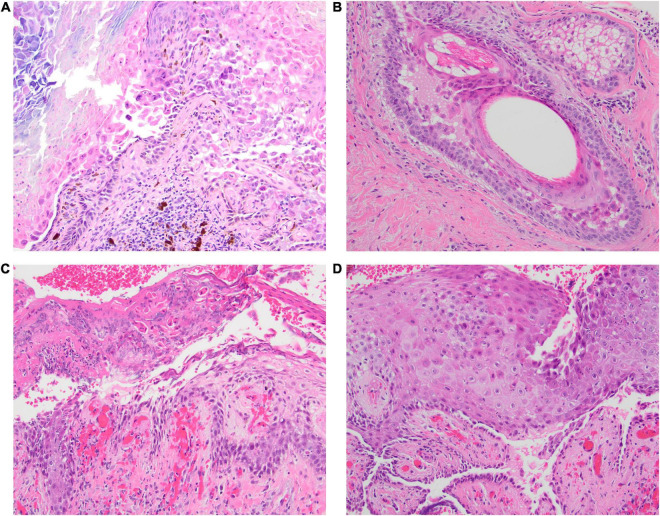
**(A–D)** The initial biopsy taken from the scalp in Case 1 was interpreted on H&E (40x) to be consistent with Darier disease since the biopsy demonstrated acantholysis with prominent superficial dyskeratosis. Subsequent biopsy with direct immunofluorescence testing confirmed pemphigus vulgaris, without histologic evidence of pemphigus vegetans.

A scalp biopsy showed focal acantholytic dyskeratosis, favoring Darier disease (Figure 2). PAS staining showed colonization by *Malassezia* (*Pityrosporum*), and fungal culture was positive for common environmental inhabitants, including mold (*Chaetomium* and *Nigrospora*) and yeast (*Rhodotorula*).

After 2 months of being lost to follow-up, he presented with two new bullae on his wrists bilaterally. Nikolsky sign was positive. PV was suspected, and biopsy confirmed a diagnosis of PV, with DIF demonstrating intercellular epidermal (cell surface) deposition of IgG and C3. Serum IgG levels were not obtained in the setting of inconsistent care. The patient was started on fluocinolone oil, IVIG, prednisone, and mycophenolate mofetil (MMF) for his pemphigus and sertaconazole therapy for the superimposed colonization with *Rhodotorula*. His condition improved on this regimen. One year later, he experienced a flare involving his upper and lower extremities, as well as mouth soreness. A repeat biopsy of his scalp, which appeared similar to the initial presentation, was consistent with PV, revealing a suprabasal blister within the epidermis and acantholytic cells. The tissue culture of his scalp was negative for fungus. His blisters resolved with a course of rituximab and IVIG; he was subsequently lost to follow-up. Healing of the keratotic lesions lagged behind blister re-epithelialization but, eventually, all lesions resolved.

## Case 2

A 39-year-old African American woman with a history of schizophrenia and illicit drug use was hospitalized for a 6-month history of rash unresponsive to medical therapy. Two months prior, she was diagnosed with disseminated MRSA cellulitis and treated with ceftriaxone, rifampin, and vancomycin. Additionally, she received nystatin and fluconazole for indeterminate hyperkeratotic lesions on the scalp. On physical exam, scattered large, brown verrucous plaques with associated alopecia were present on her scalp. Her forehead, periorbital areas, cheeks, nasolabial folds, and chin were affected with non-continuous areas of erosion and brown-gray plaques with thick scale. Superficial erosions with crust and flaccid blisters were noted on her back, trunk, and extremities with a positive Nikolsky sign. She had no ocular and oral mucosal involvement.

A biopsy of her upper back revealed an intracorneal blister containing acantholytic cells and a mixed-cell infiltrate; DIF was positive for epidermal intercellular C3 and IgG. A dermatopathologist interpreted these findings to be most consistent with PV. She was prescribed prednisone 80 mg daily, MMF 1,000 mg two times daily, and clobetasol 0.05% ointment two times daily at the time of her discharge.

**FIGURE 3:**
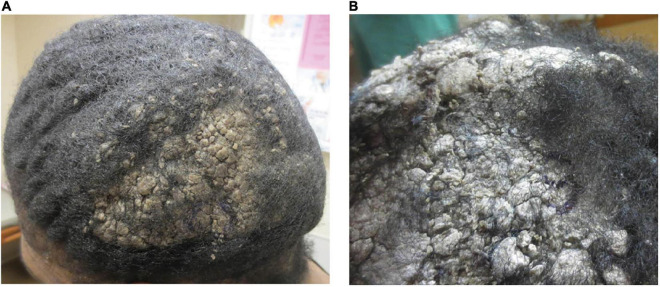
**(A)** Mounded and refractory keratoses presenting as exuberant scale on the scalp (Case 2). **(B)** Close-up image of a plaque.

At 1 month follow-up, she reported improvement of previously involved sites but noted persistent lesions on her scalp. Multiple plaques with mounded scale and a verrucous appearance were noted on her scalp (Figure 3), while her trunk and extremities showed predominately post-inflammatory hyperpigmentation. Given her persistent keratotic scalp lesions, a biopsy was obtained and confirmed PV. A pemphigus panel was positive for autoantibodies to Dsg1 and Dsg3. A tissue culture of her scalp to rule out fungal infection was negative. Subsequent evaluations were sporadic and consisted primarily of hospital consults.

## Case 3

A 65-year-old African American man with a history of hypertension, thyroid cancer status post total thyroidectomy, and colon cancer status post hemicolectomy was referred to dermatology for a 1.5-month history of a pruritic rash with tender erosions on the neck, chest, and scalp. Prior treatments included clotrimazole 1% cream, miconazole cream, triamcinolone 0.1% ointment, and a 10-day course of sulfamethoxazole/trimethoprim. On physical exam, he had multiple superficial ulcers and erosions with purulent drainage and honey-colored crust on his chest and neck. One intact flaccid bulla was documented on his chest. His occipital scalp had multiple scaly patches with hemorrhagic and honey-colored crust. No oral lesions were noted at this time. HSV and VZV cultures were negative. Two punch biopsies of his upper chest and gram stain from the bulla were obtained. An empiric course of cephalexin and mupirocin ointment was initiated for possible infection.

Biopsy revealed eosinophilic spongiosis; DIF showed weak intercellular epidermal IgG reactivity. Serologic studies were positive for autoantibodies to Dsg 1 and Dsg 3, confirming the diagnosis of PV. Gram stain was unrevealing. The patient was empirically started on clobetasol ointment but continued to have worsening symptoms of new tender bullae on his back and a new onset of mouth soreness. He was subsequently started on prednisone 80 mg daily but experienced palpitations, weakness, and shortness of breath after his first dose. He was admitted to the hospital and diagnosed with acute diastolic congestive heart failure. During his hospitalization, repeat DIF testing showed epidermal intercellular IgG and C3 reactivity, favoring PV, and he was discharged on prednisone 40 mg daily, MMF 1,000 mg two times daily, and niacinamide 500 mg three times daily.

He then completed 4 weekly infusions of rituximab at a dose of 375 mg/m^2^ BSA and experienced resolution in his symptoms. Subsequently, he was able to taper and ultimately discontinue prednisone and MMF therapy. He received a second course of rituximab for a flare 2 years later.

**FIGURE 4:**
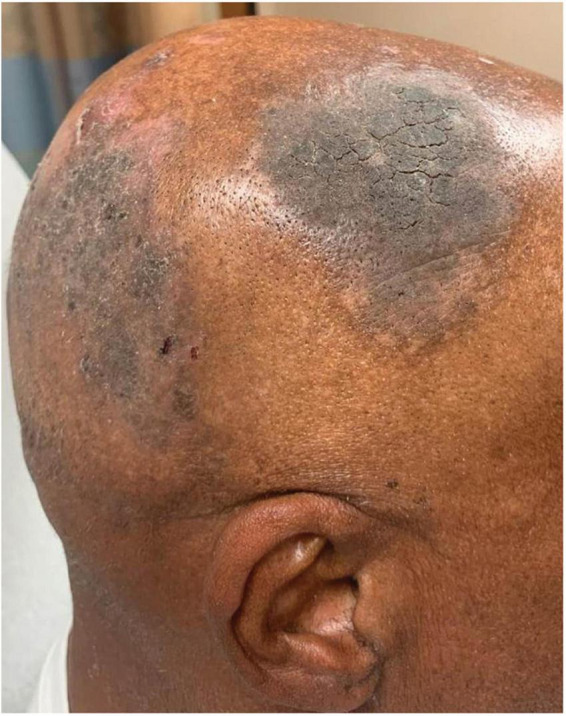
Mounded and refractory keratoses as an early presentation of pemphigus vulgaris recurrence. In this patient (Case 3), there were no blisters present on the body or scalp.

Three years later, he returned with new pruritic plaques on his scalp that were dissimilar from his previous PV lesions. A physical exam revealed circular gray, mounded plaques affecting his vertex scalp (Figure 4). No other lesions were identified on his body, and Nikolsky sign was negative. A punch biopsy from the forehead showed only non-specific spongiosis, and concurrent DIF from his scalp was negative. Three weeks later, a repeat biopsy of his scalp confirmed PV. Unfortunately, the patient was unable to tolerate a full repeat course of rituximab due to rash, pruritus, and jaw and neck soreness. The patient was restarted on a short course of MMF, but soon after discontinued due to intolerance (nausea, vomiting, and diarrhea), and thereafter, started on clobetasol solution and IVIG. His plaques and pruritus continued to persist on this regimen. The decision was made to attempt another course of rituximab. He successfully completed a full rituximab course, which resolved his plaques and symptoms.

## Case 4

A 48-year-old Hispanic man was referred to dermatology for the evaluation of a 6-month history of multiple enlarging crusted plaques on his scalp with associated pruritus and tenderness. Previous treatments included clobetasol solution, coal tar shampoo, and surgical removal. The initial lesion began as a singular red bump that progressively grew. After attempted excision, multiple lesions recurred. The bacterial culture obtained on initial evaluation was positive for staph aureus and the patient was treated with cephalexin. He was then started on a regimen of clobetasol solution, 2% salicylic acid shampoo, and mineral oil, without improvement. Initial biopsy revealed suprabasilar acantholysis with prominent dyskeratosis, which was interpreted by a dermatopathologist to be most consistent with warty dyskeratoma.

On physical exam, he had multiple verrucous or mounded keratoses over his posterior and anterior scalp with large areas of scarring alopecia. Additional biopsies of his scalp were reviewed by a dermatopathologist, again favoring warty dyskeratoma, although it was noted that the concomitant, direct immunofluorescence (DIF) revealed weak intercellular and junctional IgG and C3, evoking consideration of pemphigus. Repeat DIF testing from another scalp biopsy demonstrated similar equivocal results. A diagnosis of PV was finally established after indirect immunofluorescence (IIF) returned positive for intercellular substance (1:160); serum IgG levels were not obtained. He was then started on oral prednisone and intravenous immunoglobulin (IVIG) was thereafter added. He achieved complete resolution after 6 months and has remained in remission off therapy for 13 years.

## Discussion

It is increasingly apparent that photographs of even common dermatologic diseases in the skin of color are not well-represented in dermatology education or literature (6, 7). Despite PV being one of the most well-characterized diseases in dermatology, the clinical presentation of mounded and refractory keratoses (MARK) remains to be described. Our case series highlights the diagnostic challenges of atypical presentations of dermatologic conditions in Black and Hispanic patients. It is important that such cases are presented to the dermatologic community to reduce misdiagnosis and iatrogenic harm.

In MARK, the scalp may be a preferential site of involvement, as all patients in our case series presented with mounded, exuberant, scaly plaques at this site. However, most patients had similar lesions on their trunks as well, albeit not as severe. Of note, histologic evaluation, both hematoxylin and eosin (H&E) and DIF, of these lesions can be misleading. In our case series, initial biopsies were either inconclusive or in Cases 1 and 4, favored a different diagnosis altogether, namely, Darier disease (keratosis follicularis) or warty dyskeratoma given the shared presence of suprabasilar acantholysis (8, 9). Providers should be mindful that acantholysis can be more easily visualized in hair follicle epithelia, therefore, adequate depth in biopsy is essential and clinical-pathological correlation should always be considered. Additional clinical experience will be required to determine if the prominent dyskeratosis that we encountered in our cases typifies the histologic appearance of MARK involving the scalp. The authors would like to emphasize that MARK is a mnemonic for this interesting presentation of pemphigus. Although it might be reasonable to categorize MARK as a subtype of pemphigus vegetans, it is not the goal of the authors to present MARK as a subtype of pemphigus. Interestingly, upon review of the pemphigus vegetans literature, we could identify two prior cases that appeared to have lesions consistent with MARK (10, 11). However, strictly speaking, MARK does not overlap with pemphigus vegetans as it is most commonly defined now. In contrast to MARK, the skin lesions of pemphigus vegetans begin as erosions and are characterized by papillomatous vegetations that are most prominent on flexural surfaces (4, 12). Furthermore, none of the cases presented here exhibited eosinophilic microabscesses, a feature that has been described to be characteristic of pemphigus vegetans (13).

Mounded and refractory keratoses can be an important indicator of PV in both newly diagnosed and established patients. In two of the four patients described here, pemphigus was not a leading clinical or histologic differential diagnosis until the disease progressed to exhibit classic PV blisters at other body sites. While the scalp is not the most common presenting location for PV, 16–60% of patients with PV are reported to have scalp involvement (14). Scalp involvement in pemphigus has also been associated with more resistant and severe disease (15). In our series, mounded and refractory keratoses were the initial sign of PV either at initial presentation or at the time of relapse. For example, in three of the four patients, the recurrence of MARK was the major clinical finding indicating PV relapse. Notably, in Case 3, MARK was the only manifestation of disease recurrence in a patient who had previously presented with exclusively classic PV lesions. Thus, mounded and refractory keratoses can occur at any time point. Although these lesions were most prominent on the scalp, some patients had lesions with similar morphology noted on the face, trunk, and extremities. Interestingly, all patients in our case series were Fitzpatrick skin phototypes 4–6. It is still too soon to know if this is typical, but providers should be mindful of this presentation in the patients with skin of color.

In summary, PV may present atypically on the scalp and other body sites as MARK, especially in skin of color. In our case series, this clinical presentation resulted in an initial misdiagnosis. Diagnostic testing for PV in these patients, including histopathology, DIF, IIF, and ELISA may initially be misleading. Repeat testing can be performed if clinical suspicion is high. More specifically, a resemblance to acantholytic dyskeratosis may be characteristic of MARK on scalp biopsy. Awareness and recognition of MARK may aid providers in the diagnosis of PV and may reduce delays in starting appropriate therapy.

## Data availability statement

The original contributions presented in this study are included in the article/supplementary material, further inquiries can be directed to the corresponding author.

## Ethics statement

Ethical review and approval was not required for the study on human participants in accordance with the local legislation and institutional requirements. The patients/participants provided their written informed consent to participate in this study. Written informed consent was obtained from the individual(s) for the publication of any potentially identifiable images or data included in this article.

## Author contributions

SL and MF drafted the manuscript. All authors contributed to the study’s conception, design, material preparation, data collection, analysis, critically reviewed the manuscript, read, and approved the final manuscript.
